# Climate change and recent genetic flux in populations of *Drosophila robusta*

**DOI:** 10.1186/1471-2148-5-4

**Published:** 2005-01-06

**Authors:** Max Levitan, William J Etges

**Affiliations:** 1 Center for Anatomy and Functional Morphology and Department of Human Genetics, Box 1007, Mount Sinai School of Medicine, 1 Gustave L. Levy Place, New York, NY 10029, USA; 2 Department of Biological Sciences, University of Arkansas, Fayetteville, AR 72701, USA

## Abstract

**Background:**

Studied since the early 1940's, chromosomal polymorphisms in the deciduous woods species *Drosophila robusta *have been characterized by well-defined latitudinal, longitudinal, and elevational clines, but – until at least ten years ago – stable, local population frequencies. Recent biogeographical analyses indicate that *D. robusta *invaded North America from southeast Asia and has persisted in eastern temperate forests for at least 20–25 my without speciating. The abundant chromosome polymorphisms found across the range of *D. robusta *are thus likely to be relatively ancient, having accumulated over many well known climatic cycles in North America. Sufficient long-term data are now available such that we can now gauge the rate of these evolutionary changes in natural populations due to environmental change.

**Results:**

Recent local collections have revealed significant changes in the frequencies of several chromosomal forms. New data presented here extend the range of these changes to six states, three in the northeastern United States and three west of the Mississippi River. These data reinforce recent directional changes in which the frequencies of three gene arrangements have reached percentage levels typical of distant southern populations consistent with regional climatic changes. Another gene arrangement has been steadily decreasing in frequency at a number of the sites studied. Meteorological records from 1945 to 2003 indicate temperature increases at all study sites, particularly average minimum air temperatures.

**Conclusions:**

Observation of parallel genetic flux suggests that these long-term temporal frequency shifts in widely disparate populations of *D. robusta *are evolutionary responses to environmental change. Since these chromosomes are known to be sensitive to ambient temperature, regional climatic shifts associated with global warming are likely to be responsible.

## Background

In recent years, numerous publications – over 50 pages of them in current listings of the bibliographic search engine Medline – have detailed changes in biological systems and organisms that appear associated with the climatic changes commonly referred to as global warming (e.g., [1-5], dealing with observed or impending shifts in the habitats of various organisms). Few studies, however, have documented long-term changes in the *genetic structure *of species populations on a regional scale, necessary for understanding the microevolutionary consequences of global change [6]. In the genus *Drosophila*, comprised of over 1500 species [7], only four species have received such attention. While some long term increases have been documented in the frequencies of chromosomal gene arrangements Standard (*ST*), Tree Line (*TL*), and Pike's Peak (*PP*) in *Drosophila pseudoobscura *in the western United States and Canada over ca 40 years [8-10] and of several arrangements in *Drosophila melanogaster *in Japan [11], little is known of the causes for these changes.

Orengo and Prevosti [12] first suggested that climatic warming was responsible for long-term temporal changes in the frequencies of certain gene arrangements in European populations of *D. subobscura*. Later, Rodriguez-Trelles and co-workers [13-15] and Sole', et al. [16] convincingly demonstrated that climatic changes, including global warming, were likely the driving force of microevolutionary changes in these populations.

Recently, significant changes have also been documented in the chromosomal variation of *D. robusta *Sturtevant, some of them possibly attributable to global warming [17-19]. Here we describe additional data that underscore the variety of historical changes being experienced by populations of this species. Clearly, the chromosomal polymorphisms in *D. robusta *are also dynamic, and when compared with the considerable geographical and experimental data available for this species collected over the last 60 years, strongly implicate regional climatic changes as a cause for these temporal frequency shifts.

## Results

**Figure 1:**
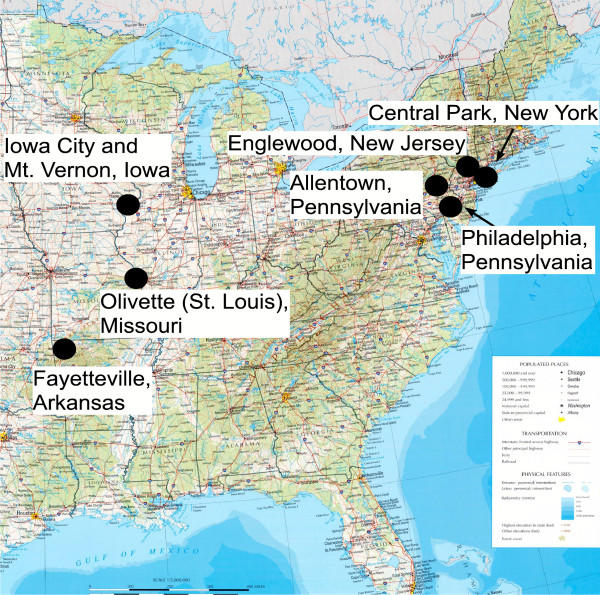
Map of the eastern United States showing the locations of collecting sites in this study. This modified map is used by permission of the University of Texas Libraries, the University of Texas at Austin.

Regional patterns of climate change were revealed from ANCOVA analysis of temperature and precipitation data from 1945 – 2003. No long term tends were detected for precipitation except for a significant increase in Central Park, NY (data available from the authors). Significant long-term temperature changes were apparent at all six 2003 study sites (Fig. 1; plus Philadelphia, last studied in 2002) for the three available temperature indicators: average monthly minimum temperature (*MINTMP*), average monthly temperature (*AVETMP*), and average maximum monthly temperature (*MAXTMP*; all 3 ANCOVA models, P < 0.0001). *AVTEMP *and *MAXTMP *varied significantly from site to site in different years, but *MINTMP *significantly increased over the 58-year period (P < 0.0001) with no heterogeneity among sites. Thus, the most consistent change in climate across sites in this study was due to significant increases in minimum monthly temperatures.

**Figure 2:**
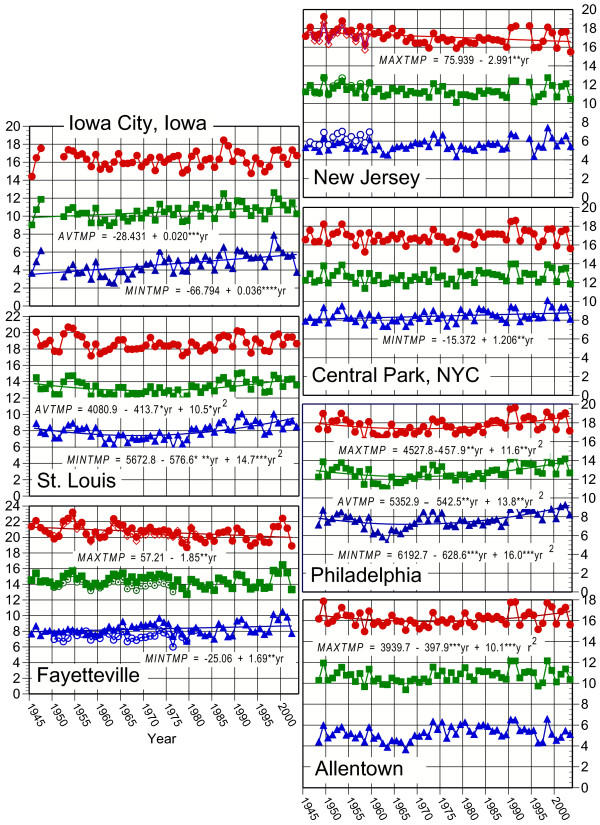
Temperature data for each site in this study plotted from 1945 to 2003. Regression lines and equations are shown only for statistically significant trends in mean maximum monthly temperature (*MAXTMP*), mean monthly temperature (*AVTMP*), and mean minimum monthly temperature (*MINTMP*) at each site. Significance and sign of the regression coefficients are indicated (*P < 0.05, ** P < 0.01, *** P < 0.001, **** P < 0.0001). Y axes are in degrees Centigrade. Replicate data plotted for Fayetteville and New Jersey show incomplete secondary weather station data at sites closer to the locations where flies were collected. See text for details.

Evidence for site-specific temperature increases from 1945 – 2003 was evident for these three temperature indicators (Fig. 2). Five of the seven sites showed linear or curvilinear increases in *MINTMP*, usually starting in the 1970's and extending until 2000. In contrast, both Fayetteville and Englewood, NJ have experienced significant temporal decreases in *MAXTMP *(with a concurrent significant increase in *MINTMP *in Fayetteville). Causes for these decreases are obscure, but in New Jersey may be due to the exaggerated temperature fluctuations in the early 1990's (as well as missing data for 1992 and 1993, Fig. 2).

**Figure 3:**
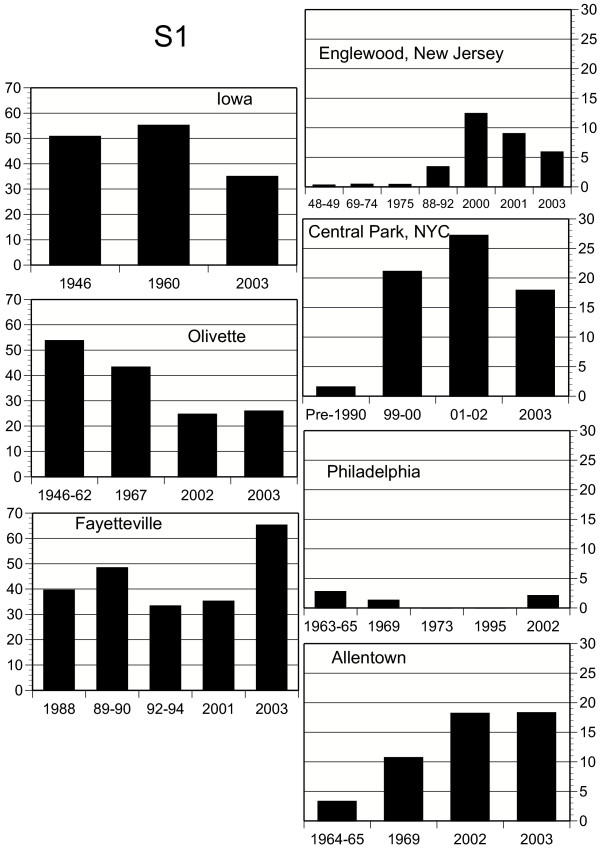
Frequencies (in percent) of *D. robusta X*-chromosome arrangement combination *S1 *in the seven study localities, including Philadelphia [18]. When frequencies across years were statistically homogeneous, they were combined within each locality. Y axes for eastern populations reflect the much lower frequencies for S1 in that part of the species range.

**Figure 4:**
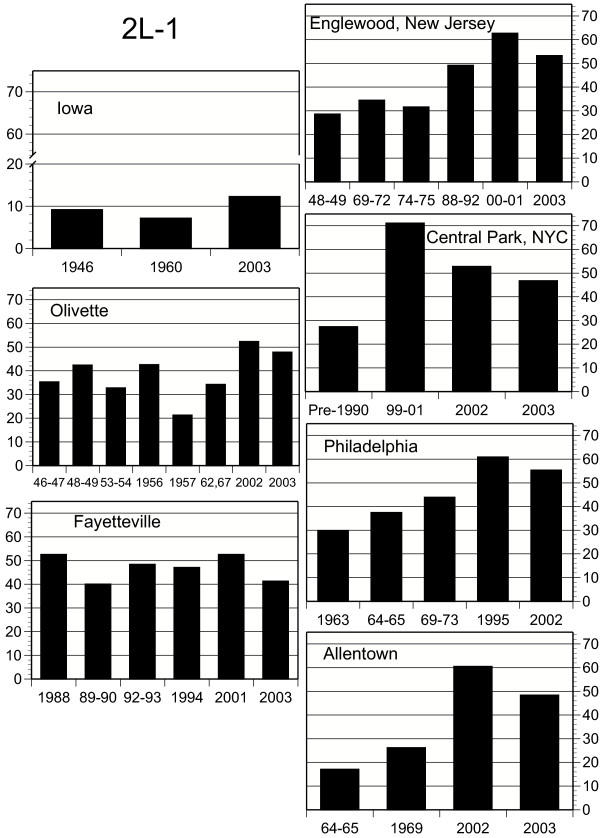
Frequencies (in percent) of *D. robusta *gene arrangement 2L-1 in the seven study localities, including Philadelphia [18]. See Fig. 3 for details.

**Figure 5:**
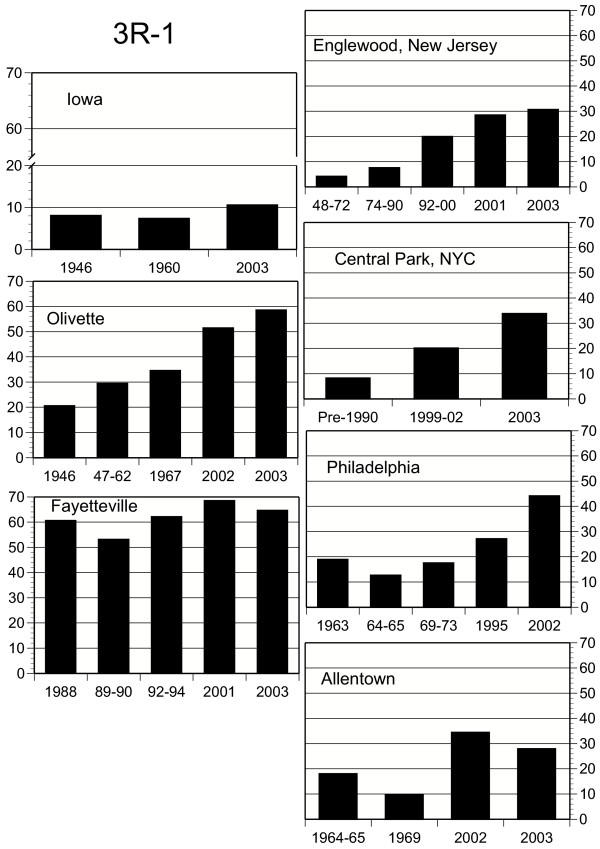
Frequencies (in percent) of *D. robusta *gene arrangement 3R-1 in the seven study localities, including Philadelphia [18]. See Fig. 3 for details.

Concurrent with these temperature shifts, chromosome elements in populations of *D. robusta *show systematic temporal frequency changes (all frequency data are available from the authors). X chromosome combinations 1S and S1 show substantial frequency changes with time, with S1 doing so in a site-specific manner (Fig. 3). Frequencies of combination 1S have decreased over time, and are negatively correlated with increasing temperatures (Tables 1, 2). Increases in S1 are only marginally correlated with temperature, but this may be due to other factors, including its low frequency in eastern populations (Fig. 3). Gene arrangements 2L-1 and 3R-1 have also increased in frequency since 1945 (Fig. 4, 5; Tables 1, 2), and the changes are significantly site-specific as indicated by the significant year × site interactions. While temporal changes in 2L-1 and 3R-1 are correlated (r = 0.419, P = 0.0016, n = 54), only those of 3R-1 are significantly correlated with increasing temperatures (Fig. 5; Table 2).

**Table 1:** ANCOVA results for temporal changes in the frequencies of several X chromosome arrangement combinations and autosomal inversions across the seven collecting sites in this study. r^2 ^is an estimate of the proportion of the total variance explained by the model used.

	X chromosome combination 1S				
Source	df	Type III SS	F Value	Pr > F	r^2^

Model	13	1.151	50.31	< 0.0001	0.942
Year	1	0.014	5.97	0.019	
Site	6	0.207	14.93	< 0.0001	
Year*Site	6	0.199	14.35	< 0.0001	

					

	X chromosome combination S1				

Source	df	Type III SS	F Value	Pr > F	r^2^

Model	13	2.589	50.94	< 0.0001	0.943
Year	1	0.000	0.02	0.894	
Site	6	0.206	8.79	< 0.0001	
Year*Site	6	0.197	8.39	< 0.0001	

					

	Gene arrangement 2L-1				

Source	df	Type III SS	F Value	Pr > F	r^2^

Model	13	1.317	20.55	< 0.0001	0.870
Year	1	0.038	7.66	0.0085	
Site	6	0.215	7.25	< 0.0001	
Year*Site	6	0.216	7.29	< 0.0001	

					

	Gene arrangement 3R-1				

Source	df	Type III SS	F Value	Pr > F	r^2^

Model	13	2.054	54.09	< 0.0001	0.946
Year	1	0.043	14.76	0.0004	
Site	6	0.057	3.28	0.0102	
Year*Site	6	0.060	3.40	0.0084	

**Table 2:** Pearson product-moment correlations between average monthly temperature and frequencies of several X chromosome arrangement combinations and autosomal arrangements for all seven localities studied. n = 54 for all tests.

	1S	S1	2L-1	3R-1
Average temperature for month of collection	- 0.341	0.250	0.195	0.336
	P = 0.012	P = 0.071	P = 0.163	P = 0.014

The most consistent historical changes have been gains in the frequency of arrangement 3R-1 with concomitant decreases of its allelic form, 3R (Figure 5). Coupled with the clinal tendency of 3R-1, varying between 100% in the southernmost latitudes and zero in the northernmost [20], its similar directional increases of recent years in at least four states, some far apart, and concordance with long term temperature increases indicates that 3R-1 is responding to regional climatic shifts.

The frequency of arrangement 2L-1 increased directionally in most of the same localities as 3R-1 (Figure 4). The frequency of 2L-2, which is also more common in the south than in the north, has been decreasing steadily, almost to the point of extinction, in the four northern 2003 populations. This is probably not a temperature effect, however, as in the past 2L-2 has been irregularly distributed across the southern states, rarely rising above frequencies of 20 percent, without evidence of a latitudinal cline [20-22].

Like 2L-1 and 3R-1, the frequency of gene arrangement XL (the sum of data for *SS*, *S1*, and *S2*), tends to be higher in southern populations than in northern ones [20]. Except for a few deviations in small samples, it has increased steadily – with concomitant decreases in northern arrangement XL-1 – in all the localities studied except Olivette (where it is already about 95%) and Iowa. At Englewood, for example, it rose from about 40% in 1948 to an average of 78% in 2000–2003, that of northern arrangement XL-1 falling from about 60% to about 22% in the same period. Frequencies of X chromosome combination 1S (XL-1.XR) are clearly consistent with this pattern (Tables 1, 2).

## Discussion

Studies of *Drosophila *inversion polymorphisms have now provided historical insights into environmental change. On different continents, temporal genetic changes correlated with increasing temperatures in populations of *D. subobscura *[12-16] and *D. robusta *[[17,18], this study] bear the imprint of large scale climate change. For *D. robusta*, not all temporal changes were consistent with this hypothesis. In the course of comparing plant growth in rural and urban areas in relation to ozone exposure, rural areas tended to have lower average temperatures than urban ones [23]; this may explain the smaller (or lack of) change in 3R-1 frequencies in relatively rural Fayetteville and Iowa City as compared to the more urban locales. Also, there are fewer data available for these sites collected over time spans long enough to document temporal trends. Another possibility is that the climatic changes have not been of equal intensity in all localities, as indicated by regional increases and decreases in local temperatures (Fig. 2) suggested by the site by year interactions for *AVTMP *and *MAXTMP *in the ANCOVA analyses.

Carson [24] noted that the chromosomal variation in populations of *Drosophila robusta *at Olivette, Missouri over a 10-year period was characterized by "extraordinary stability," wherein "certain frequencies may shift significantly as compared with the previous year, but in every case the observed frequencies approximate some previously observed level." This pattern continued until at least 1967 (Fig. 3, 4, 5). Temperature records from this period seem rather stable before significantly increasing through 2000 (*AVTMP *and *MINTMP *second order regression slopes are both positive; Fig 2). Similar stability, frequency changes of less than 10 percent, has also been recorded near Blacksburg, Virginia from 1950 to 1962 (M. Levitan, unpublished data) and in northeastern New Jersey from 1948 until at least 1975 (Fig. 3, 4, 5).

By contrast, every population sampled in 2003 evidenced at least one significant change of chromosomal polymorphism frequency compared to the numbers in the same area ten or more years previously, with indication that the changes in at least three are part of similar, if not identical, directional historical processes. Therefore, documentation of temporal genetic changes in these populations requires at least 10 to 20 years of comparative data.

Influences of natural selection due to ambient temperature variation on frequencies of these gene arrangements and X chromosome associations have been demonstrated in laboratory experiments [24,25], and inferences from latitudinal and multiple elevational clines [26-31]. Frequencies of arrangements XL-1 and 2L-3 are clearly associated with cooler temperatures at higher elevations and latitudes, and 2L-1 increases in frequency in the laboratory under warmer temperatures. Carriers of 2L-3 have shorter egg to adult development times expressed in cooler temperatures, explaining increases in this gene arrangement with elevation and latitude [26].

Evidence of strong natural selection maintaining earlier observed genetic stability has also come from perturbation experiments in the wild [20]. At four time periods, large numbers of flies carrying X-chromosome combinations *S2 *and *22*, autosomal combination 2L-1.2R-1 (*11*), and 3R-1 from South Carolina, Alabama, and Mississippi were released in midsummer in the Englewood, NJ woods. In other years the released flies carried XL-1, 2L-3, and 3R from Minnesota and Michigan. Despite evidence of hybridization of the introduced flies with the local population, in each case the following spring saw return to frequencies of the previous year.

According to Wright [32], "The alternative to natural selection in a changing environment is, as noted by Dobzhansky [33], the emergence of superior genetic systems" in the gene arrangements that have been increasing in frequency. He envisioned this happening in one population by recombination in inversion homozygotes, with the new adaptive gene complex in one or a few flies spreading to other localities by "occasional very long dispersion by the wind." He conceded that such a hypothesis would be most satisfactory for cases where a very rare inversion suddenly started increasing, such as the rises of *TL *and *PP *of *D. pseudoobscura *mentioned above, since high frequencies of inversion homozygotes would undermine the structural stability of the new adaptive complex. The sudden rises of *D. robusta *X-chromosome combination *S1 *in New York and New Jersey and of *S2 *in Missouri could be cases in point, but it would not explain the near doubling of *S1 *in Arkansas from a 35% base between 2001 and 2003 nor the three most consistent directional changes, those of 3R-1, 2L-1, and XL (Figures 3, 4, 5). Indeed, the nearly simultaneous changes in places as far apart as New York and Allentown, Pennsylvania, let alone New York and Missouri, would be very unlikely to depend on chance wind-blown dispersions.

Here, causes for systematic frequency changes in widespread populations can be inferred to be a result of common environmental causes given the large amount of background data available for *D. robusta*. When compared to the inversion frequency data collected over 60 years from more than 150 natural populations [20,21] and results from numerous laboratory experiments, temporal frequency shifts across the range of *D. robusta *strongly suggest temperature variation as a likely mechanism driving microevolutionary change [17,18]. Such regional frequency shifts suggest a common response to temperature fluctuations or causes correlated with them, whether due to regional climatic changes or global warming.

## Conclusions

Although direct evidence for climatic change has been accumulating for many years, its consequences for causing evolutionary changes have only recently been observed. Chromosome polymorphisms in *Drosophila *species have been historically important genetic systems for understanding mechanisms of evolutionary change, and have now been studied long enough to begin revealing widespread, systematic temporal frequency shifts in response to environmental change. These polymorphisms thus represent excellent indicators of future climatic shifts.

## Methods

There are 14 commonly encountered gene arrangements segregating in natural populations of *D. robusta *located on five of the six arms of the 3 major chromosomes. The "Standard" arrangements were labeled for the respective chromosome arms: XL, XR, 2L, etc. Others were named in order of their discovery, e.g., XL-1, XL-2, XR-1, 2L-1 [described and configured in [20,24]].

The Standard arrangement of each arm was dubbed "S," and the other arrangements are referred to by the Arabic numerals in their names [34]. A fly with karyotype XL/XL-1, XR/XR-2, for example, would be S/1, S/2 in this notation. Depending on the linkage combination of the arrangements, it is also either *SS*/*12 *or *S2*/*1S*.

Linkage relationships are inferred from karyotypic analyses of adult males and females [35]. Female *D. robusta*, unlike many other drosophilids, quickly deplete stored sperm in the absence of remating so that wild-caught females can be despermed by repeated transfers to fresh food vials and then crossed to homokaryotypic males in a controlled fashion. Karyotypes of at least 6 larvae from these test crosses were prepared in order to infer the linkage combination of X chromosome gene arrangements. Salivary gland smears from larvae derived from matings in the wild, so-called "egg sample" data, were included when collected females did not survive the desperming transfers.

This report compares data obtained in 2003 to earlier summer collections at a number of geographically isolated populations going back in some cases to 1946: Olivette, a suburb of St. Louis, Missouri; woods alongside Route 4 in Englewood, New Jersey; Trexler Memorial Park on the western outskirts of Allentown, Pennsylvania; the North Woods of Central Park in New York City; Fayetteville, Arkansas; and woods along the Iowa River at Iowa City, Iowa.

The significance of year-to-year differences in frequency of each chromosome or chromosome arm obtained prior to 2003 was determined by G-tests [36]. Chronologically contiguous results that proved statistically homogeneous are combined in the figures. Individual X- and second-chromosome arrangements could not be tested in this way due to problems of independence.

## Analysis of meteorological data

Temperature and precipitation data were obtained online from the National Climatic Data Center  for stations with complete records extending from 1945–2003 nearest to each site. Continuous data for Fayetteville and Englewood were not available for the closest weather stations, so records from those stations with complete records were used. Correlations between temperatures from these stations were highly significant: Drake Field and the Agricultural Experiment Station for Fayetteville (r = 0.48 – 0.90, P < 0.01), Little Falls and Ridgefield for Englewood (r = 0.84 – 0.98, P < 0.0001); see Fig. 2. Analysis of covariance in PROC GLM [36] was used to test for overall significance of temperature trends and chromosome frequency changes across sites, and to evaluate regional patterns of temperature and precipitation change. Polynomial regression analyses of temperature and precipitation data with time were performed with PROC REG [37]. In all cases, linear or second order polynomial regression explained the most variation for a particular model. Pearson product-moment correlations between arcsin transformed chromosome frequencies and the average temperature of the month for each collection were calculated with PROC CORR [37]. Non-parametric correlation analyses produced equivalent results. We also assessed correlations with temperatures for the month prior to collection, and the average temperature of the 3 months prior to collection: none were significant.

## Authors' contributions

ML collected the Allentown, New York, and New Jersey flies and carried out the chromosomal analyses. WJE contributed the Fayetteville flies. Both authors contributed to planning the study, analyzing the data, and writing and revising the manuscript.
